# Volumetric Changes in the Upper Airways after Rapid and Slow Maxillary Expansion in Growing Patients: A Case-Control Study

**DOI:** 10.3390/ma13102239

**Published:** 2020-05-13

**Authors:** Valentina Lanteri, Marco Farronato, Alessandro Ugolini, Gianguido Cossellu, Francesca Gaffuri, Francesca Maria Rita Parisi, Davide Cavagnetto, Andrea Abate, Cinzia Maspero

**Affiliations:** 1 Department of Biomedical, Surgical and Dental Sciences, School of Dentistry, University of Milan, 20100 Milan, Italy; valentina.lanteri@unimi.it (V.L.); marcofarronato@msn.com (M.F.); gianguido.cossellu@unimi.it (G.C.); francesca.gaffuri@unimi.it (F.G.); francescamr.parisi@gmail.com (F.M.R.P.); davide.cavagnetto@gmail.com (D.C.); andreabate93@gmail.com (A.A.); 2 Fondazione IRCCS Cà Granda, Ospedale Maggiore Policlinico, 20100 Milan, Italy; 3 Department of Sciences Integrated Surgical and Diagnostic, University of Genova, 16132 Genova, Italy; alexugolini@yahoo.it

**Keywords:** palatal expansion technique, airway resistance, maxillary expansion, maxillary sinus, cone-beam computed tomography

## Abstract

The aim is to evaluate changes in the volume of the upper airways before and after slow maxillary expansion (SME) obtained with the flexible properties of a nickel titanium leaf spring and rapid maxillary expansion (RME) with a conventional Hyrax appliance in growing patients. The records of 1200 orthodontic patients undergoing maxillary expansion from 2018 to 2019 were analyzed; among these pre and post treatment CBCT scans of 22 patients (mean age 8.2 ± 0.6 years old) treated by SME were compared with those obtained from 22 patients (mean age 8.1 ± 0.7 years old) treated by RME banded on the second primary molars. The following inclusion criteria were used: Maxillary transverse constriction, good general health, and no previous orthodontic treatment. Volumes of nasal cavity (NCavV), nasopharynx (NsPxV), and right and left maxillary sinuses (MSV) were calculated with ITK-SNAP. Shapiro–Wilk test revealed a normal distribution of data in each group. Paired *t*-test was used for within-group comparisons and independent *t*-test for between-group comparisons. Statistically significant increases occurred in NCavV, NsPxV, and MSVs after treatment with both appliances. No statistically significant difference between the appliances occurred in NCavV, NsPxV, and MSVs. Method error was considered negligible (mean intra-operator and inter-operator intraclass correlation coefficient were 0.928 and 0.911, respectively). It appears that both appliances produce similar effects on the different segments of the upper airway tract.

## 1. Introduction

### 1.1. Background

The tooth-borne maxillary expansion is a common dentofacial orthopedic treatment for maxillary hypoplasia in children and teens [1,2,3,4,5]. This orthopedic expansion is obtained through appliances whose active part is a transversal screw that transmit a lateral force to the upper posterior teeth, inducing the split of the midpalatal suture and a certain amount of widening of circummaxillary sutures (frontomaxillary, zygomaticomaxillary, zygomaticotemporal, and pterygopalatine) [6,7].

Several studies have already observed that orthopedic expansion of the maxilla also causes an increase of the rhino-pharynx, the nasal cavity, and the paranasal sinuses air volumes [8,9,10,11,12,13]. Radiographic assessment was originally performed using bidimensional radiographs, by lateral and postero–anterior cephalograms [14]. Subjects were also examined with functional rhinomanometry and polysomnography tests and presented a significant reduction in nasal resistances and their breathing improved [15,16,17,18,19].

These studies were later repeated using volumetric imaging techniques, such as cone-beam computed tomography (CBCT) [20,21,22] and magnetic resonance imaging (MRI) [23], to overcome drawbacks of traditional cephalograms in evaluating bi-dimensionally distorted complex anatomical 3D structures. These data quantified unambiguously the actual volumetric changes of the different segments of the upper respiratory tract and helped clinicians and researchers to better understand and give reason to the improvement registered in abovementioned functional tests.

Two appliances for maxillary expansion are commonly used in clinical practice: Palatal acrylic (Haas-type) and hygienic (Hyrax) expanders. No clinically relevant difference in terms of orthopedic effects appears from available literature [24]. Tooth-borne maxillary expansion can be rapid (rapid maxillary expansion (RME)) or slow (slow maxillary expansion (SME)) depending on the activation protocol of the active part of the appliance (i.e., number of screw turns per day). RME protocols use heavy and continuous forces for shorter lapses of time, significant effects on maxillary structures occur immediately. Forces in SME protocols are intermittent and lighter, and they act for longer periods [25]. Both of them appear to have similar orthopedic effects in growing patients [25,26].

In 2013 a new expander with a small body size, and equipped with two nickel titanium leaf springs was introduced with the commercial name of Leaf Expander [27]. It is otherwise similar to a conventional Hyrax expander; it allows the release of calibrated and continuous forces to promote maxillary expansion. It was designed especially for compliance-free SME with an optimized force system [27,28]. It delivers a constant lateral force of 450 g or 900 g, depending on clinical needs, and it allows a maximum expansion of 6 or 10 mm. The appliance is engineered to deliver the first 3 mm of expansion without any activation (compressing of the leaf spring by the screw). It is supposed to be re-activated in office once a month by 10 quarter turns (1 mm of activation) until the achievement of desired expansion. It requires no compliance from patients’ parents, it is less painful compared to conventional expanders and appears to deliver a similar expansion [27,28,29,30]. Since its recent introduction in the market, limited evidence is available. However few, published data appear promising [27,28,29,30], but several aspects of patients’ biological response to this appliance still need to be investigated further.

### 1.2. Aim

The aim of the present study was to assess and compare the effect of SME obtained with the Leaf Expander and of RME obtained with a conventional Hyrax expander on the volumetric modifications assessed on CBCT scans that occur to different segments of the upper respiratory tract in patients before the growth peak.

## 2. Materials and Methods

This is a retrospective case-control study on changes of the upper airway tract before and after expansion treatment either with RME or Leaf Expander using the CBCT scans of patients treated at the Department of Biomedical Surgical and Dental Sciences of the University of Milan, Fondazione IRCCS Ca’ Granda, Ospedale Maggiore Policlinico Milan, between February 2018 and March 2019. The study protocol was approved by the Ethical Committee of the Fondazione IRCCS Ca’Granda, Ospedale Maggiore, Milan, Italy (protocol n.573/15). Signed informed consent for releasing diagnostic records for research purposes was obtained from parents of all patients included in the study.

### 2.1. Sample Selection and Inclusion Criteria

Patients’ records that had CBCT scans taken for different reasons before and after maxillary expansion were selected from the records archived at the Department of Biomedical, Surgical and Dental Sciences.

Inclusion criteria were: Good general health, no other dental issue apart from maxillary transverse hypoplasia due to maxillary constriction with intermolar width <31 mm, age between seven to nine years old; maxillary expander (RME or SME ) cemented on the upper second primary molars; and the first CBCT scan was taken no more than three months before maxillary expansion and the second one was taken after a time lapse between 10 and 14 months from appliance cementation. The reasons behind the just described time criterium for the second CBCT scan to be taken was that six months are required from the last RME activation to consolidate orthopedic results and mean comprehensive treatment duration (active treatment and retention period) lasts 10 months according to literature [16,31] and that the mean treatment duration with the Leaf Expander lasts 9 months [27]. The reason behind the age group selection of patients is twofold. Patients between seven and nine years old usually have the second primary molars that offer a free anchorage to the appliance and according to morphometric three-dimensional studies appear to present the least possible expansion of nasal and paranasal sinuses during growth [21], hence measured changes can be allegedly attributed to orthopedic effect of the appliance and not to spontaneous enlargement during growth. 

A total of 1200 medical records were analyzed. Twenty-two patients treated with SME met the inclusion criteria and formed the case group. The sample was composed of 9 males (mean age 7.9 ± 0.4 years old) and 13 females (mean age 8.2 ± 0.6 years old). Mean distance between CBCT scans was 11.4 months.

The control group of the present study consisted of 22 patients who underwent rapid maxillary expansion with Hyrax expander and met all inclusion criteria. The sample was composed of 11 males (mean age 8.4 ± 0.9 years old) and 11 females (mean age 8.1 ± 0.7 years old). Mean distance between CBCT scans was 10.8 months. 

### 2.2. CBCT Examination and Data Processing

All CBCT scans were obtained using the iCAT ® FLX V-17 Series cone-beam dental-imaging system (1910 N. Penn Road, Hatfield, PA 19440) with the head in natural head position (that is with the Frankfurt plane parallel to the ground), a voxel size of 0.4, a slice thickness of 0.4mm, and different field of view dimension depending on clinical needs. The raw data were then exported, reconstructed and converted into digital imaging and communications in medicine (Dicom3) file format. The Dicom3 files were then analyzed with ITK-SNAP software, version 2.2.0 (www.itksnap.org), an open access popular library image analysis algorithm funded by the US National Library of Medicine [32]. Its validity in assessing the volume of upper airway tract and subdivide the 3D virtual model of airway space into different anatomical and functional segments after maxillary expansion has already been validated by Almuzian et al. [33,34], whose protocol has been followed in the present study. 

Patients’ airways have been segmented into four parts: Nasal cavity (NCavV), nasopharynx (NsPxV), and left and right maxillary sinuses (MSV).

ITK-SNAP allows the exclusion of potential masking changes of the adjacent or remote airway spaces. A fixed threshold was chosen rather than an interactive one to eliminate operator bias of custom threshold selection [35]. 

Each segment was defined and outlined in the sagittal and coronal dimensions for the software to calculate the volume as all CBCT were taken in natural head position with the Frankfurt plane parallel to the ground. 

On sagittal slices the midsagittal plane was identified and the nasal cavity was defined by the following points and lines: The caudal limit by the palatal plane that is the line extending from the posterior nasal spine (PNS) to the anterior nasal spine (ANS); the ventral limit by the line connecting ANS to the tip of the nasal bone (TNB) and then to nasion point (N); the cranial limit by the line from N to sella (S); and the dorsal limit by the line from S to PNS. On coronal slices the nasal cavity was identified by drawing a line all the way along the outline of the nasal cavity. 

The volume of the nasopharynx on sagittal slices was defined ventrally by the line from PNS to S, dorsally by the line from S to the tip of the odontoid process (CV2tp), and caudally by the line from CV2tp to PNS. 

The maxillary sinuses can be outlined individually by drawing a line on the sagittal midline section, including the maxillary first molar furcation, around the superior, inferior, medial, and lateral walls of each maxillary sinus. This process is repeated on a coronal section showing the widest area of the maxillary sinus.

**Figure 1:**
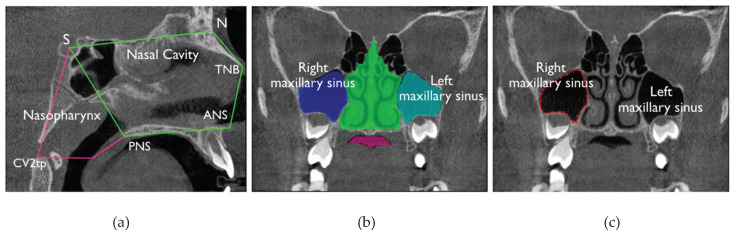
Visual explanation of planes and points used on the different cone-beam computed tomography (CBCT) axis for ITK-SNAP to perform volume segmentation of the respiratory segments analyzed: Nasal cavity, maxillary sinuses, and rhynopharynx. (**a**) sagittal view; (**b**,**c**) coronal views.

**Figure 2:**
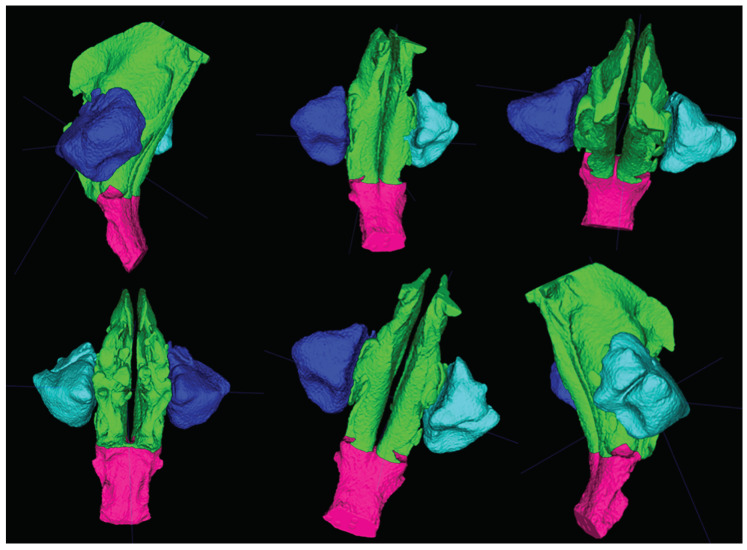
Multiple view of studied reconstructed volumes segmented into the different portions analyzed in this study: Nasal cavity, maxillary sinuses, and rhynopharynx.

The definitions of anatomic areas and the obtained 3D volume renderings are shown in Figure 1 and Figure 2.

### 2.3. Maxillary Expansion

Both appliances were anchored on primary second molars with a glass-ionomer orthodontic luting cement (Multi-Cure; Unitek, Monrovia, CA, USA) [36].

Patients treated with SME followed the protocol designed especially for maxillary expansion with the leaf expander appliance which delivers a constant lateral force of 450 g.

Parents of patients treated with RME were instructed to perform a turn of the Hyrax screw twice a day (half a millimeter per day) for the first seven days and then revalued by the orthodontist that would either decide to stop or continue to activate the appliance. Eventual additional activations were checked weekly and continued till reach of desired expansion.

In both appliances a ligature wire was used to block the hyrax screw or the Nichel Titanium (NiTi) spring when patients presented dental overcorrection, that is when palatal cusps of the upper first permanent molars occluded on the edge of the lingual side of vestibular cusps of mandibular first permanent molars.

### 2.4. Statistical Analysis

Sample size was calculated a priori to obtain a statistical power of the study greater than 0.85 at an alpha of 0.05, by G*Power (version 3.1.9.4, Franz Faul, Universitat Kiel, Kiel, Germany) using the mean values and standard deviations of maxillary molar expansion (MME) after RME treatment found by Cerruto et al. [19].

The sample size calculation indicated that 18 participants were needed to reach an 85% power of the analysis and to perform statistically meaningful comparisons. 

IBM SPSS Statistics version 25.0 software (IBM Co., Armonk, NY, USA) was used for statistical comparisons. Shapiro–Wilk test showed that data were normally distributed in all groups, therefore parametric tests were used to perform within and between groups comparisons. Descriptive statistics was therefore expressed as mean value ± standard deviation and 95% confidence interval (CI).

Independent *t*-test was used to compare pre-treatment right and left MSV in each group. Paired *t*-test was used to perform within group comparisons for all the measured parameters.

Independent *t*-test was used to perform between groups comparison on volumetric changes occurred after maxillary expansion between the SME and RME groups for all the variables considered.

All volumetric measurements were carried out by one senior examiner (F.G.). All volumetric measurements calculations were repeated for 7 days on 10 randomly selected CBCT by the same observer (F.G.) and by the second observer (F.P.) to determine the method error. Intra- and inter-observer differences were statistically analyzed by paired Wilcoxon test. In addition, the intra-class correlation coefficients (ICC) were calculated. A p value < 0.05 was set as statistically significant.

## 3. Results

The ICC values for the intra- and inter-observer agreement for volumetric measurements were 0.928 (95% CI: 0.867–0.950; p < 0.001) and 0.911 (95% CI: 0.828–0.945; p < 0.001). Overall, the method error was considered negligible.

Descriptive statistic is reported in Table 1. 

Comparisons between right and left MSVs in each group before treatment are presented in Table 2.

Comparisons of the upper airway and MSVs before and after SME and RME treatment are presented in Table 3. A statistically significant increases were found for NCavV, NsPxV, and MSVs after treatment with both SME and RME. 

**Figure 3:**
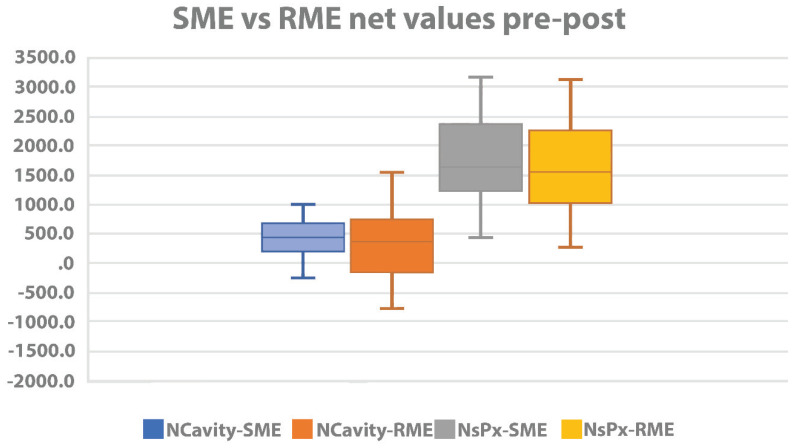
Boxplot comparing net values of mean, interquartile range, and data range of nasal cavity volume and mean, interquartile range, and data range of nasopharyngeal volume before and after treatment with SME or RME.

Treatment comparisons between SME and RME groups are presented in Table 3. No statistically significant difference has been noticed comparing volumetric changes of NCavV, NsPxV, and MSVs and (Table 4, Figure 3).

## 4. Discussion

The aim of the present study was to assess whether a newly designed SME could produce orthopedic effects similar to RME on the volumes of the upper pharyngeal airway tract and on MSVs. Dentists, especially the ones practicing orthodontics, have been interested in changes occurring to the upper airway tract from a long time. As reported by several authors, nasal breathing and low terminal respiratory resistances are crucial to promote a correct development of craniofacial structures [37,38,39,40,41,42,43,44]. The influence that orthodontic and dentofacial orthopedic treatments exerts on the respiratory function has been extensively studied [15,21,45,46]. Maxillary expansion is a commonly performed treatment for solving one of the most common orthodontic issue of the developing child: Maxillary hypoplasia.

Apart from orthopedically solving conditions of severe crowding and/or dento-alveolar incongruences, it is known to increase upper airway space and reduce respiratory resistances [47,48,49]. Maxillary expansion is capable to induce changes in the craniofacial structures like increasing nasal cavity volume and reducing nasal resistances to airflow that can result even in a reduction of apnea hypopnea index (AHI) of patients affected by obstructive sleep apnea [50]. Previous studies evaluated changes occurring in the upper airway and maxillary sinus volumes after RME treatment. Moreover the effects of RME on pharyngeal airways and maxillary sinus have been investigated in previous studies [51,52,53] with two-dimensional radiographs [54] and three- dimensional CBCT scans [55,56,57,58]. However, no study, as yet, evaluates whether volumetric changes occurs to the upper airway of patients treated with this newly designed SME.

Basciftci et al. demonstrated that rapid maxillary expansion produces an increase in nasal floor width near the mid-palatal suture and nasal cavity [59]; besides, the lateral walls of the nasal cavity, pushed laterally from the opening of the midpalatal suture, cause an augmentation of the inter-nasal volume. Doruk et al. [60] also demonstrated a statistically significant increase in the volume of nasal cavity after rapid maxillary expansion using CT and confirmed functional breathing benefits using acoustic rhinometry. These results were confirmed later by Gorgulu et al. [1] analyzing the effect of rapid maxillary expansion on nasal volume with CBCT.

Buck et al. reported in a systematic review that maxillary expansion causes the total volume of the upper airway to increase regardless of the type of expander appliance used [15]. Lanteri et al. reported the tested appliance to be effective in solving maxillary hypoplasia in growing patients. The main advantages brought by this appliance into clinical practice include ease of use, no compliance needed from the patient nor from his family, and obtaining a slow mid-palatal suture opening with the use of predetermined and constant forces [27,28,61].

Moreover, it was demonstrated by Ugolini et al. [62] constant forces exerted by the NiTi spring during maxillary expansion allow to avoid most of the pain that occurs at the beginning of the activation of a RME.

In the current study, a statistically significant increase in NCavV and NsPxV after SME and RME treatment was found, with a similar percentage of increase between the two appliances. When SME and RME treatment were compared, there was no statistically significant difference in NCavV and NsPxV between the two appliances. The results obtained in the present study confirmed that the effect of the leaf expander on pharyngeal airway was comparable to those obtained with a conventional RME.

However, the effect of RME on pharyngeal airway volume in literature is still a matter of debate. Smith et al. [63] investigated volumetric changes of the pharyngeal airway after RME treatment during the adolescence. In their study, statistically significant increases in nasopharyngeal airway volume after RME treatment were noticed, similar to the present study. Conversely, Ribeiro et al. [64] evaluated nasopharyngeal changes with CBCT after RME and reported that only oropharyngeal airway experienced a significant increase, while the nasopharyngeal airway did not. Zhao et al. [65] reported no significant differences both in the oropharynx and in the rhinopharynx. The apparent contradictions between these studies could be owed to different methods such as the absence of standardized positioning of the head and tongue and different segmentation protocols. [65,66].

In the present study, the changes in right and left MSVs for SME and RME were also evaluated. In the pre-treatment comparison, maxillary sinuses appeared perfectly symmetrical in both groups demonstrated by the absence of statistically significant differences between right and left MSV. In both treatment groups, MSV had a statistically significant increase after treatment on both sides. When the effects of the appliances were compared, there was no significant difference between the two groups.

As for pharyngeal airway, MSV have also been evaluated in literature with different results.

Darsey et al. [55] found that there was no significant increase of the MSV after RME treatment. Garrett et al. [67] found that maxillary sinus width was reduced after RME treatment, and thus should MSV. Pangrazio–Kulbersh et al. [68] investigated the nasopharyngeal airway and MSV changes after RME appliance with three-dimensional CBCT images. Their results were similar to those of the present study, with a significant increase of the MSV after treatment. The differences between them may be explained by the same reasons as pharyngeal airway mixed results.

In the current study ITK-SNAP was used. The key feature of the software is the existing facilities to segment and navigate through the volumetric data set in three planes of space with a linked cursor system that allows tracking of a single voxel. The automatic segmentation process allows construction of the main 3D virtual surface, while the semi-automatic segmentation allows fine-tuned segmentation to identify the most appropriate border between neighboring structures [69,70].

The main limitations of the present study are a relatively small sample (albeit sufficient for inferential statistics consideration) and the difficulty of retrieving multiple scans of a patients over time to allow a better understanding of volumetric longitudinal changes over time. The constant development of radiation-free imaging, like magnetic resonance imaging, will hopefully help in this particular problem. Another limitation of this study could by the age group that was selected to be analyzed. However, pre-pubertal age is the most indicated age to perform maxillary expansion as, according to Baccetti et al. [67], subjects undergoing RME during this particular stage of development before the peak of growth show greater and more stable skeletal changes in all involved structures. As the sample in the present study analyzes patients before the growth spurt further studies with slow maxillary expansion after the pubertal peak of growth are needed.

## 5. Conclusions

The results of this research confirm the effectiveness of SME in treating maxillary hypoplasia in the mixed dentition. This treatment appeared effective in increasing pharyngeal airway and MSV in patients with maxillary hypoplasia. No statistically significant difference was noted when comparing its results with the one obtained using a conventional Hyrax-RME. Slow maxillary expansion appears to be a valid, painless, and no compliance alternative to RME regarding evaluated variables.

## Figures and Tables

**Table 1:** Descriptive statistics: Mean value ± standard deviation (SD) and confidence interval (CI) for both groups at each timepoint.

Volumes	SME		RME	
	Mean ± SD	CI 95% Lower Limit-Upper Limit	Mean ± SD	CI 95% Lower Limit-Upper Limit
NCavV T0	1271 ± 364	1109.7–1432.2	1216 ± 715	1126.3–1496.9
NCavV T1	1701 ± 399	1524.4–1878.1	1715 ± 518	1453.2–1959.8
ΔNCavV	430 ± 331	283.4–577.2	306 ± 614	160.6–629.1
NsPxV T0	3663 ± 821	3299.5–4027.1	3568 ± 855	3762.6–4280.5
NsPxV T1	5406 ± 821	5042.0–5770.1	5254 ± 812	4558.7–5529.0
ΔNsPxV	1743 ± 680	1441.2–2044.3	1684± 810	1537.9–1976.7
R-MSV T0	8806 ± 1102	8297.5–9289.0	8546 ± 713	8230.1–8862.2
R- MSV T1	9358 ± 938	8942.2–9774.2	9314 ± 988	8901.5–9753.3
ΔR- MSV	553 ± 932	156.5–973.4	768 ± 1181	260.9–1301.6
L- MSVT0	8575 ± 983	8139.3–9011.4	9063 ± 1101	8535.7–9451.4
L- MSV T1	9140 ± 1225	8596.4–9682.7	9888 ± 1287	9317.4–10458.5
ΔL MSV	564 ± 1110	72.0–1056.5	825 ± 1145	422.9–1366.0

Abbreviations: NCavV = nasal cavity volume; NsPxV = nasopharynx volume; MSV = maxillary sinus volume; R = right; and L = left.

**Table 2:** Descriptive statistics (mean ± standard deviation) and independent *t*-test comparing right and left side of maxillary sinus volume before expansion treatment in each group.

Volumess	SME		RME	
	Mean ± SD	p Value	Mean ± SD	p Value
L- MSVT0	8575 ± 983	0.23	9063 ± 1101	0.092
R- MSV T1	8806 ± 1102		8546 ± 713	

* p value < 0.05 were considered to indicate statistical significance.

**Table 3:** Descriptive statistics (mean ± standard deviation), confidence interval, and paired *t*-test comparing upper airway volumes before (T1) and after (T2) in both groups.

Volumes	Mean ± SD(ΔT1-T0)	CI 95% Lower Limit-Upper Limit	p Value	% Increase
NCavV T1-T0 (RME)	306 ± 614	160.6-629.1	0.020 *	30.1
NCavV T1-T0 (SME)	430 ± 331	283.4-577.2	< 0.01 *	33.9
NsPxV T1-T0 (RME)	1684± 810	1537.9-1976.7	< 0.01 *	42.4
NsPxV T1-T0 (SME)	1743 ± 680	1441.2-2044.3	< 0.01 *	47.6
R- MSV T1-T0 (RME)	768 ± 1181	260.9-1301.6	0.005 *	9.1
R- MSV T1-T0 (SME)	553 ± 932	156.5-973.4	0.011 *	6.4
L- MSV T1-T0 (RME)	825 ± 1145	422.9-1366.0	0.002 *	9.9
L- MSV T1-T0 (SME)	564 ± 1110	72.0-1056.5	0.027 *	6.6

* p value < 0.05 were considered to indicate statistical significance.

**Table 4:** Descriptive statistics (mean ± standard deviation) and independent *t*-test between volumetric changes (ΔT1-T0) obtained with slow maxillary expansion (SME) and rapid maxillary expansion (RME).

Volumes	SME (ΔT1-T0)	RME (ΔT1-T0)	p Value
NCavV	430 ± 331	306 ± 614	0.385
NsPxV	1743 ± 680	1864 ± 810	0.12
R- MSV	553 ± 932	768 ± 1181	0.49
L- MSV	564 ± 1110	825 ± 1145	0.44

* p value < 0.05 were considered to indicate statistical significance.
